# Analytical sphere–thin rod interaction potential

**DOI:** 10.1140/epje/s10189-025-00480-9

**Published:** 2025-04-07

**Authors:** Junwen Wang, Shengfeng Cheng

**Affiliations:** 1 Department of Mechanical Engineering, Virginia Tech, Blacksburg, VA 24061 USA; 2 Center for Soft Matter and Biological Physics, Virginia Tech, Blacksburg, VA 24061 USA; 3 Macromolecules Innovation Institute, Virginia Tech, Blacksburg, VA 24061 USA; 4 Department of Physics, Virginia Tech, Blacksburg, VA 24061 USA

## Abstract

**Abstract:**

A compact analytical form is derived through an integration approach for the interaction between a sphere and a thin rod of finite and infinite lengths, with each object treated as a continuous medium of material points interacting by the Lennard-Jones 12-6 potential and the total interaction potential as a summation of the pairwise potential between material points on the two objects. Expressions for the resultant force and torque are obtained. Various asymptotic limits of the analytical sphere–rod potential are discussed. The integrated potential is applied to investigate the adhesion between a sphere and a thin rod. When the rod is sufficiently long and the sphere sufficiently large, the equilibrium separation between the two (defined as the distance from the center of the sphere to the axis of the rod) is found to be well approximated as $a+0.787\sigma$, where *a* is the radius of the sphere and $\sigma$ is the unit of length of the Lennard–Jones potential. Furthermore, the adhesion between the two is found to scale with $\sqrt{a}$.

**Graphic abstract):**

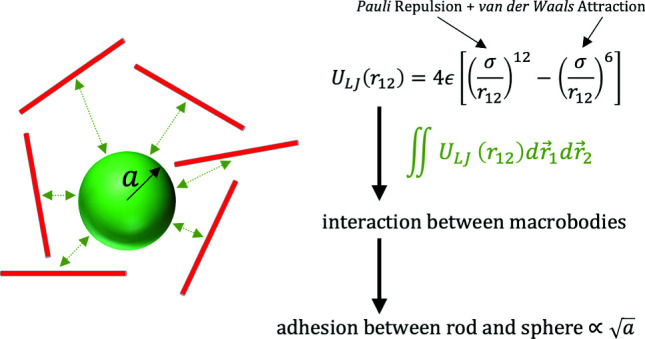

**Supplementary Information:**

The online version contains supplementary material available at 10.1140/epje/s10189-025-00480-9.

## Introduction

The Lennard–Jones (LJ) 12-6 potential is one of the most widely used functional forms to represent interatomic and intermolecular interactions [1, 2]. As a natural extension, integrated LJ potentials between condensed bodies with various geometrical shapes are used in a wide range of computational studies and theoretical analyses due to their simplicity [3, 4]. In the so-called Hamaker approach, the integrated potential between two objects is obtained by summing (or integrating) the potential between material points, of which the two objects consist, in a pairwise manner [5, 6]. To enhance the applicability of such potentials, the parameters (e.g., the Hamaker constant setting the interaction strength) in an integrated potential can be tuned to match realistic cases. Integrated forms of the LJ 12-6 potential have been derived for a few spherical and planar geometries [3, 7–10]. The integrated potential between two spheres has been implemented in the Large-scale Atomic/Molecular Massively Parallel Simulator (LAMMPS) [11, 12]. The LJ potential has also been integrated between a point particle and a plane or a half-space and implemented in LAMMPS as various wall potentials [13].

In addition to spheres, cylindrical objects are abundant in synthetic and natural systems [14], including liquid crystal molecules, colloidal nanorods, nanopillars, carbon nanotubes, nanowires, biofilaments (e.g., microtubules), and rod-shaped virus (e.g., tobacco mosaic virus) and microorganisms (e.g., *Escherichia coli* bacteria). In general, it is more challenging to integrate the LJ potential for cylinders. Most attempts have been made in the studies of carbon nanotubes [15–21] and some are limited to the van der Waals (vdW) attraction only. The full LJ 12-6 potential was integrated by de Rocco and Hoover for two thin rods in either collinear or parallel configurations [7]. Hamady et al. derived an analytical expression for the interaction between a nanorod and a three-dimensional half-space filled with LJ point particles [22]. An approximate form of the integrated LJ potential was proposed by Vesely for two sticks in more general settings [4]. Recently, full analytical forms for the integration of both $1/r^6$ attraction and $1/r^{12}$ repulsion have been obtained by Wang et al. for thin LJ rods in arbitrary three-dimensional arrangements [6], by means of Ostrogradsky’s integration method [23, 24].

To theoretically and computationally study mixtures of rods and spheres, it is important to include their mutual interactions. Through computer simulations based on the Gay-Berne and LJ potentials, Antypov and Cleaver showed that the phase behavior of rod-sphere mixtures is sensitive to the strength and symmetry of the rod-sphere interaction [25]. Jadrich and Schweizer found that the strength and the spatial range of interparticle attraction are key control parameters determining the relationship between kinetic arrest, connectivity percolation, structure, and phase separation in rod-sphere mixtures [26]. Liu et al. showed that directional attraction can lead rod-sphere mixtures to form ordered phases [27]. Opdam et al. showed that including excluded volume interactions in the free volume theory is critical to predict the phase behavior and stability of rod-sphere mixtures [28, 29]. They further developed a general theoretical framework to describe thermodynamic properties such as the multiphase coexistence behavior of multicomponent mixtures of hard colloids, including spheres, rods, and plates [30]. Therefore, it is valuable to develop algebraic expressions for rod-sphere interaction potentials with solid physical foundations.

Several results have been previously reported in the literature on rod-sphere interactions. Rosenfeld and Wasan obtained the exact result on the nonretarded vdW attraction between a sphere and an infinite cylinder with a finite radius by integrating the $1/r^6$ potential [31]. Kirsch later confirmed this result and further obtained the compact expression for the retarded case (i.e., the integrated form of the $1/r^7$ attractive potential) [32]. Gu and Li studied both retarded and nonretarded vdW interactions between a sphere and a cylinder with a finite length and cross section by combining analytical and numerical integrations [33]. Montgomery et al. calculated the dispersion forces for several nontraditional geometries, including the case of a sphere and an infinite cylinder [34]. He et al. obtained an analytical expression for the vdW attraction between a nanoparticle and a nanorod with a finite length and radius under certain approximations [35]. However, a compact form of the integrated LJ potential between a sphere and a rod has been elusive.

Here we report a fully analytical form of the interaction potential between a sphere and a thin rod with finite or infinite lengths in an arbitrary configuration by integrating the LJ 12-6 potential. The sphere is treated as a continuum and the thin rod is modeled as a material line consisting of LJ point particles. The integrated sphere–rod potential is expressed as an analytical function form, and the associated expressions for force and torque are also presented. These forms can be used in theoretical analyses and computational modeling of sphere–rod mixtures [25–30]. As an application, the integrated sphere–rod potential is used to investigate the adhesion between a sphere and a thin rod and an interesting scaling dependence of the adhesion on the sphere size is found.

## Theoretical model of sphere–rod interaction

### Integrated sphere–point potential

The LJ 12-6 potential between two point particles reads

1 $$\begin{aligned} U_\text {LJ}(r) = 4\epsilon \left[ \left( \frac{\sigma }{r}\right) ^{12} - \left( \frac{\sigma }{r}\right) ^6 \right] ~, \end{aligned}$$

where $\epsilon$ is an energy scale, $\sigma$ is a length scale, and *r* is the distance between the two particles. The LJ 12-6 potential has been integrated between two spheres, each of which is considered a uniform distribution of LJ particles at a number density of $1.0\sigma ^{-3}$ [3]. By reducing one sphere into a point particle, the integrated sphere–point potential is obtained [12]. In general, for a point particle and a sphere consisting of LJ particles at a number density of $\rho _s$, the sphere–point potential reads

2 $$\begin{aligned} U_\text {SP}(r)= &   \frac{2\rho _s a^3\sigma ^6 A_{cs}}{9} \nonumber \\  &   \left[ \frac{\left( 5 a^6 +45 a^4 r^2 + 63 a^2 r^4 +15 r^6\right) \sigma ^6}{15 \left( r^2-a^2\right) ^9} \right. \nonumber \\  &   \left. - \frac{1}{\left( r^2-a^2\right) ^3}\right] ~, \end{aligned}$$

where *r* is the center-to-center distance between the point particle and the sphere, *a* is the radius of the sphere, and $A_{cs} = 24\pi \epsilon$ is a Hamaker constant setting the interaction strength. Clearly, $r>a$ is required in Eq. (2) as the point particle cannot overlap with the sphere.

### Integrated sphere–rod potential

**Fig. 1:**
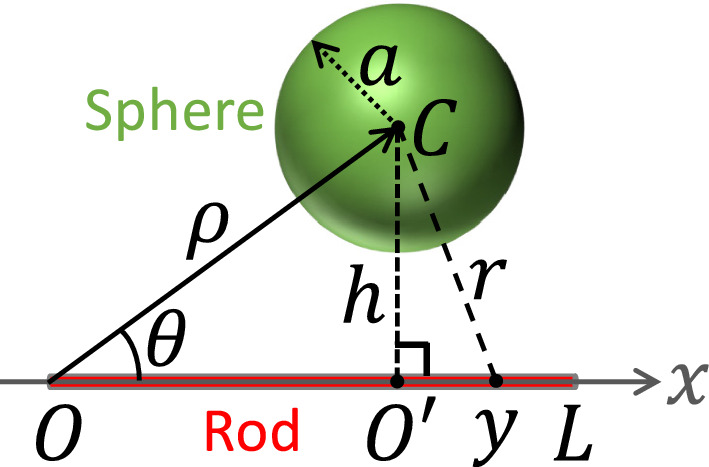
A polar coordinate system describing a general configuration of a sphere and a thin rod

The sphere–point potential can be further integrated to obtain the interaction between a sphere and a thin rod. A general sphere–rod configuration is shown in Fig. 1. By setting the *x*-axis along the central axis of the rod and choosing one end of the rod as the origin, we can build a polar coordinate system with the center of the sphere located at $(\rho ,\theta )$. It is always possible to build such a frame with $0 \le \theta \le \pi$. The interaction potential between the sphere and the rod can then be denoted as a function, $W(\rho ,\theta )$, which represents the integrated form of the sphere–point interaction potential in Eq. (2).

3 $$\begin{aligned} W(\rho ,\theta )= &   \lambda \int _{0}^{L} U_\text {SP}(r)\vert _{r=\sqrt{x^2 + \rho ^2 -2 x \rho \cos \theta }}~dx~, \end{aligned}$$

where *L* is the length of the rod, $\lambda$ the line number density of LJ material points of which the rod consists.

With a change of variables, $y=x-\rho \cos \theta$ and $h = \rho \sin \theta$, the geometric meanings of which are sketched in Fig. 1, the integral in Eq. (3) can be transformed into

4 $$\begin{aligned} W(\rho ,\theta )= &   \lambda \int _{0}^{L} U_\text {SP}(r)\vert _{r=\sqrt{y^2 + h^2}}~dx~. \end{aligned}$$

Considering the form of $U_\text {SP}(r)$ in Eq. (2), Eq. (4) involves an integral of rational functions, which can be evaluated using Ostrogradsky’s method [23, 24]. The result can be written as

5 $$\begin{aligned} W(\rho ,\theta ) = \frac{2\lambda \rho _s a^3\sigma ^6 A_{cs}}{9} \left[ G(L,\rho ,\theta ) - G(0,\rho ,\theta )\right] , \end{aligned}$$

where the function $G(x,\rho ,\theta )$ is given by

6 $$\begin{aligned}  &   G(x,\rho ,\theta ) = \sigma ^6 \left[ \frac{8 a^6 y}{15 \left( h^2-a^2\right) \left( h^2+y^2-a^2\right) ^8} \right. \nonumber \\  &   -\frac{4 y \left( 4 a^6-9 a^4 h^2\right) }{35 \left( h^2-a^2\right) ^2 \left( h^2+y^2-a^2\right) ^7} \nonumber \\  &   +\frac{y \left( 11 a^6-9 a^4 h^2+63 a^2 h^4\right) }{105 \left( h^2-a^2\right) ^3 \left( h^2+y^2-a^2\right) ^6} \nonumber \\  &   + y \left( 16 a^6+216 a^4 h^2+378 a^2 h^4+105 h^6\right) \nonumber \\  &   \times \left( \frac{1 }{1050 \left( h^2-a^2\right) ^4 \left( h^2+y^2-a^2\right) ^5} \right. \nonumber \\  &   +\frac{3 }{2800 \left( h^2-a^2\right) ^5 \left( h^2+y^2-a^2\right) ^4} \nonumber \\  &   + \frac{1 }{800 \left( h^2-a^2\right) ^6 \left( h^2+y^2-a^2\right) ^3} \nonumber \\  &   +\frac{1 }{640 \left( h^2-a^2\right) ^7 \left( h^2+y^2-a^2\right) ^2} \nonumber \\  &   \left. + \frac{3 }{1280 \left( h^2-a^2\right) ^8 \left( h^2+y^2-a^2\right) } \right) \nonumber \\  &   \left. + \frac{3 \left( 16 a^6+216 a^4 h^2+378 a^2 h^4+105 h^6\right) }{1280 \left( h^2-a^2\right) ^{17/2}} \right. \nonumber \\  &   \quad \left. \times \arctan \left( \frac{y}{\sqrt{h^2-a^2}}\right) \right] \nonumber \\  &   - \left[ \frac{y}{4 \left( h^2-a^2\right) \left( h^2+y^2-a^2\right) ^2} \right. \nonumber \\  &   + \frac{3 y}{8 \left( h^2-a^2\right) ^2 \left( h^2+y^2-a^2\right) } \nonumber \\  &   \left. + \frac{3}{8 \left( h^2-a^2\right) ^{5/2}} \arctan \left( \frac{y}{\sqrt{h^2-a^2}}\right) \right] ~. \end{aligned}$$

Here, the repulsive and attractive components of the interaction are clearly separated.

Equation (6) holds as long as $h>a$, i.e., when the *x*-axis in Fig. 1 is outside the sphere. In the case of $h<a$, the sphere intersects the *x*-axis (i.e., the line along the central axis of the rod). The argument of the arctangent function in Eq. (6), $y/\sqrt{h^2-a^2}$, becomes a complex number. This is expected as the integrated sphere–rod potential should diverge when the two overlap. However, the potential should still be finite as long as the rod is outside the sphere, even when its axis intersects the sphere. Therefore, for $h<a$, the arctangent term in Eq. (6) needs to be transformed into

7 $$\begin{aligned}  &   \frac{1}{\sqrt{h^2-a^2}}\arctan \left( \frac{y}{\sqrt{h^2-a^2}}\right) \nonumber \\  &   \qquad = \frac{1}{2\sqrt{a^2-h^2}} \ln \left( \frac{\sqrt{a^2-h^2}-y}{\sqrt{a^2-h^2}+y}\right) ~. \end{aligned}$$

It is easy to show that for a rod outside the sphere with $h<a$, the argument of the logarithmic function in Eq. (7) is negative for $-\rho \cos \theta \le y \le L - \rho \cos \theta$. Therefore, directly using Eq. (7) still yields a complex value for $G(x,\rho ,\theta )$. However, since only $G(L,\rho ,\theta ) - G(0,\rho ,\theta )$ enters the integrated potential, the relative term involves the following expression

8 $$\begin{aligned}  &   \ln \left( \frac{\sqrt{a^2-\rho ^2\sin ^2\theta }-L+\rho \cos \theta }{\sqrt{a^2-\rho ^2\sin ^2\theta }+L-\rho \cos \theta }\right) \nonumber \\  &   \qquad - \ln \left( \frac{\sqrt{a^2-\rho ^2\sin ^2\theta }+\rho \cos \theta }{\sqrt{a^2-\rho ^2\sin ^2\theta }-\rho \cos \theta }\right) \nonumber \\  &   \quad = \ln \left( \frac{a^2-\rho ^2-L\sqrt{a^2-\rho ^2\sin ^2\theta } + L\rho \cos \theta }{a^2-\rho ^2+L\sqrt{a^2-\rho ^2\sin ^2\theta } + L\rho \cos \theta } \right) ~.\nonumber \\ \end{aligned}$$

The argument of the logarithmic function in the last line of Eq. (8) is positive and the function value is thus real.

To make it clear, in the case of $h<a$, the integrated potential can be explicitly written as

9 $$\begin{aligned} W(\rho ,\theta )|_{\rho \sin \theta <a}= &   \frac{2\lambda \rho _s a^3\sigma ^6 A_{cs}}{9} \left[ Q(L,\rho ,\theta ) - Q(0,\rho ,\theta ) \right. \nonumber \\  &   \left. + P(\rho , \theta )\right] ~, \end{aligned}$$

with

10 $$\begin{aligned} Q(x,\rho ,\theta )  &   = \sigma ^6 \left[ \frac{8 a^6 y}{15 \left( h^2-a^2\right) \left( h^2+y^2-a^2\right) ^8} \right. \nonumber \\  &   \quad -\frac{4 y \left( 4 a^6-9 a^4 h^2\right) }{35 \left( h^2-a^2\right) ^2 \left( h^2+y^2-a^2\right) ^7} \nonumber \\  &   \quad +\frac{y \left( 11 a^6-9 a^4 h^2+63 a^2 h^4\right) }{105 \left( h^2-a^2\right) ^3 \left( h^2+y^2-a^2\right) ^6} \nonumber \\  &   \quad + y \left( 16 a^6+216 a^4 h^2+378 a^2 h^4+105 h^6\right) \nonumber \\  &   \quad \times \left( \frac{1 }{1050 \left( h^2-a^2\right) ^4 \left( h^2+y^2-a^2\right) ^5} \right. \nonumber \\  &   \quad +\frac{3 }{2800 \left( h^2-a^2\right) ^5 \left( h^2+y^2-a^2\right) ^4} \nonumber \\  &   \quad + \frac{1 }{800 \left( h^2-a^2\right) ^6 \left( h^2+y^2-a^2\right) ^3} \nonumber \\  &   \quad \left. \left. +\frac{1 }{640 \left( h^2-a^2\right) ^7 \left( h^2+y^2-a^2\right) ^2} \right. \right. \nonumber \\  &   \quad \left. \left. + \frac{3 }{1280 \left( h^2-a^2\right) ^8 \left( h^2+y^2-a^2\right) } \right) \right] \nonumber \\  &   \quad - \left[ \frac{y}{4 \left( h^2-a^2\right) \left( h^2+y^2-a^2\right) ^2} \right. \nonumber \\    &   \quad \left. + \frac{3 y}{8 \left( h^2-a^2\right) ^2 \left( h^2+y^2-a^2\right) } \right] , \end{aligned}$$

and

11 $$\begin{aligned} P(\rho ,\theta )= &   \left[ \frac{3\sigma ^6 \left( 16 a^6+216 a^4 h^2+378 a^2 h^4+105 h^6 \right) }{2560 \left( a^2- h^2\right) ^{17/2}} \right. \nonumber \\  &   \left. - \frac{3}{16 \left( a^2 - h^2\right) ^{5/2}} \right] \nonumber \\  &   \times \ln \left( \frac{a^2-\rho ^2-L\sqrt{a^2-h^2} + L\rho \cos \theta }{a^2-\rho ^2+L\sqrt{a^2-h^2} + L\rho \cos \theta } \right) ~,\nonumber \\ \end{aligned}$$

where $$y=x-\rho \cos \theta $$ and $$h=\rho \sin \theta $$.

In the case of $h=a$, i.e., when the *x*-axis in Fig. 1 is tangent to the sphere, the integrated potential between the sphere and the rod can be easily evaluated to be

12 $$\begin{aligned} W(\rho ,\theta )|_{\rho \sin \theta = a}= &   \frac{2\lambda \rho _s a^3\sigma ^6 A_{cs}}{9} \left[ \frac{1}{5y^5}-\frac{\sigma ^6}{15} \left( \frac{128a^6}{17y^{17}} \right. \right. \nonumber \\  &   + \frac{72a^4}{5y^{15}} + \frac{108a^2}{13y^{13}} \nonumber \\  &   \left. \left. + \frac{15}{11y^{11}}\right) \right] ^{y= L-\rho \cos \theta }_{y= -\rho \cos \theta }~. \end{aligned}$$

It can be shown that Eq. (12) is the asymptotic form of Eqs. (5) or (9) in the limit of $h=a$, except for a singular term that depends only on *h* and is therefore canceled during the subtraction process in Eqs. (5) or (9). The continuity of the analytical expressions for the integrated sphere–rod potential at $h>a$ [Eq. (5)], $h=a$ [Eq. (12)], and $h<a$ [Eq. (9)] are explicitly demonstrated in the Supplementary Information.

For an infinite rod, the integrated sphere–rod potential can be written as

13 $$\begin{aligned} W(\rho ,\theta )|_{L=\infty }= &   \frac{2\lambda \rho _s a^3\sigma ^6 A_{cs}}{9} \lim _{L\rightarrow \infty }\left[ G(L,\rho ,\theta ) \right. \nonumber \\  &   - G(0,\rho ,\theta ) + G(L,\rho , \pi -\theta ) \nonumber \\  &   \left. - G(0,\rho ,\pi -\theta ) \right] ~. \end{aligned}$$

Note that $G(0,\rho ,\theta )=-G(0,\rho ,\pi -\theta )$ and

14 $$\begin{aligned}  &   \lim _{L\rightarrow \infty } G(L,\rho ,\theta ) \nonumber \\  &   \quad = \lim _{L\rightarrow \infty } G(L,\rho ,\pi -\theta ) \nonumber \\  &   \quad = \frac{3 \pi \sigma ^6 \left( 16 a^6+216 a^4 h^2+378 a^2 h^4+105 h^6\right) }{2560 \left( h^2-a^2\right) ^{17/2}} \nonumber \\  &   \qquad - \frac{3\pi }{16 \left( h^2-a^2\right) ^{5/2}}~. \end{aligned}$$

As expected, the integrated LJ potential between a sphere of radius *a* and an infinite thin rod depends only on *h*, the distance between the center of the sphere and the central axis of the rod, and can be written as $W(h) \equiv W(\rho ,\theta )|_{L=\infty }$, where

15 $$\begin{aligned} W(h)= &   \frac{\pi \lambda \rho _s a^3\sigma ^6 A_{cs}}{3} \nonumber \\  &   \quad \times \left[ \frac{ \sigma ^6 \left( 16 a^6+216 a^4 h^2+378 a^2 h^4+105 h^6\right) }{640 \left( h^2-a^2\right) ^{17/2}} \right. \nonumber \\  &   \qquad \left. - \frac{1}{4 \left( h^2-a^2\right) ^{5/2}} \right] ~. \end{aligned}$$

Obviously, in this case $h>a$ is required. The force between the sphere and the rod can easily be computed from $F (h) = -\text {d}W(h)/\text {d}h$. For example, the magnitude of the attractive component is

16 $$\begin{aligned} \frac{5\pi \lambda \rho _s a^3\sigma ^6 A_{cs}}{12} \frac{h}{\left( h^2-a^2\right) ^{7/2}}~, \end{aligned}$$

which is identical to the thin rod limit of the result obtained by Rosenfeld and Wasan [31].

In the limit of $a\rightarrow 0$ under the constraint of $\rho _s\frac{4\pi }{3}a^3 = 1$, the sphere is reduced to a point particle and the integrated potential in Eq. (15) is reduced to

17 $$\begin{aligned} W(h)|_{a\rightarrow 0} = \frac{\lambda \sigma ^6 A_{cs}}{16 h^5} \left( \frac{21}{32}\frac{\sigma ^6}{h^{6}} - 1\right) ~, \end{aligned}$$

which is the integrated LJ potential for a point particle at a distance *h* from an infinite rod.

**Fig. 2:**
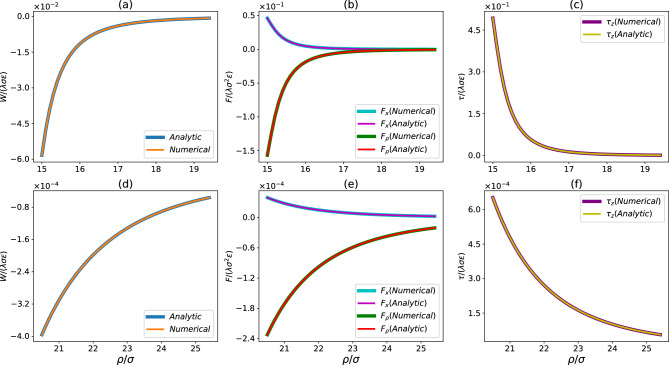
Comparison of analytical and numerical results on (**a**) and (**d**) integrated potential (*W*), (**b**) and (**e**) force components ($F_x$ and $F_\rho$), and (**c**) and (**f**) torque on the rod ($\tau$) vs. $\rho$. The results are for a sphere with $a = 10\sigma$ and a thin rod with $L=5\sigma$ at $\theta = \pi /6$. The top row is for the case with $\rho \sin \theta <a$ while the bottom row is for $\rho \sin \theta >a$

### Force and torque in sphere–rod interaction

Using the coordinate system defined in Fig. 1, together with three unit vectors, ${\textbf {n}}_x$, ${\textbf {n}}_\rho$, and ${\textbf {n}}_\theta$ along the *x*, $\rho$, and $\theta$ axes, respectively, the force on the sphere can be computed from the integrated potential, $W(\rho ,\theta )$, as

18 $$\begin{aligned} {\textbf {F}}_\text {S}= &   -\nabla W(\rho ,\theta ) \nonumber \\= &   -\frac{\partial W}{\partial \rho } {\textbf {n}}_\rho - \frac{1}{\rho } \frac{\partial W}{\partial \theta } {\textbf {n}}_\theta ~. \end{aligned}$$

Since

19 $$\begin{aligned} {\textbf {n}}_\theta = -\frac{1}{\sin \theta } {\textbf {n}}_x + \frac{\cos \theta }{\sin \theta } {\textbf {n}}_\rho ~, \end{aligned}$$

the force on the sphere can also be written as

20 $$\begin{aligned} {\textbf {F}}_\text {S} = \frac{1}{\rho \sin \theta }\frac{\partial W}{ \partial \theta } {\textbf {n}}_x - \left( \frac{\partial W}{\partial \rho } + \frac{\cos \theta }{\rho \sin \theta }\frac{\partial W}{ \partial \theta } \right) {\textbf {n}}_\rho ~. \end{aligned}$$

The force on the rod is ${\textbf {F}}_\text {R} = -{\textbf {F}}_\text {S}$ from Newton’s third law.

To compute the torque on the rod, we need to consider the interaction between the sphere centered at $(\rho ,\theta )$ and an infinitesimal segment of length *dx* on the rod, which is given by $w(x,\rho ,\theta ) \equiv \lambda U_\text {SP}(r) dx$, where $r=\sqrt{x^2 + \rho ^2 - 2 x \rho \cos \theta }$. The full sphere–rod potential, $W(\rho ,\theta )$, is obtained by integrating $w(x,\rho ,\theta )$ over *x* (see Eq. (3)). The potential, $w(x,\rho ,\theta )$, satisfies the following identity,

21 $$\begin{aligned} x\left( \frac{\partial w}{\partial \rho } + \frac{\cos \theta }{\rho \sin \theta } \frac{\partial w}{\partial \theta } \right) = \frac{1}{\sin \theta } \frac{\partial w}{\partial \theta }~. \end{aligned}$$

This identity holds as long as *w* is a function of *r* only with $r=\sqrt{x^2 + \rho ^2 - 2 x \rho \cos \theta }$.

The torque on the rod, with respect to its center, can be computed as

22 $$\begin{aligned} \varvec{\tau }_{\text {R}z} = \int (x-l){\textbf {n}}_x\times {\textbf {f}}_{\text {R}\rho } d x~. \end{aligned}$$

Here $l=L/2$ and ${\textbf {f}}_{\text {R}\rho }$ is the $\rho$-component of the force exerted on the infinitesimal segment of the rod by the sphere, which reads

23 $$\begin{aligned} {\textbf {f}}_{\text {R}}= &   \nabla w(x, \rho ,\theta ) \nonumber \\= &   - \frac{1}{\rho \sin \theta }\frac{\partial w}{ \partial \theta } {\textbf {n}}_x + \left( \frac{\partial w}{\partial \rho } + \frac{\cos \theta }{\rho \sin \theta }\frac{\partial w}{ \partial \theta } \right) {\textbf {n}}_\rho ~.\nonumber \\ \end{aligned}$$

Completing the calculation in Eq. (22) with the help of Eq. (21) and noting that $W(\rho ,\theta ) = \int w(x,\rho ,\theta )dx$, we arrive at the final expression of the torque on the rod,

24 $$\begin{aligned} \varvec{\tau }_\text {Rz} = \left[ -\frac{L}{2} \sin \theta \frac{\partial W}{\partial \rho } + \left( 1- \frac{L}{2\rho } \cos \theta \right) \frac{\partial W}{\partial \theta } \right] {\textbf {n}}_z~, \end{aligned}$$

where ${\textbf {n}}_z \equiv \frac{1}{\sin \theta } {\textbf {n}}_x\times {\textbf {n}}_\rho$. The torque is 0 for $\theta =0$ and $\pi$, where the central axis of the rod passes through the center of the sphere. Furthermore, when $\rho = L/(2\cos \theta )$, that is, when the center of the sphere is on the perpendicular bisector of the rod, the torque on the rod is also 0. These limits are encoded in the symmetry of the sphere–rod system considered here. A similar calculation shows that the torque on the rod, with respect to its *O*-end in Fig. 1, is $\frac{\partial W}{\partial \theta } {\textbf {n}}_z$, which is an expected result.

The results on force and torque presented in this section can be used directly to describe the interaction between spheres and rods in computational modeling (e.g., molecular dynamics simulation) of their mixture. However, the results are presented in a local frame defined in Fig. 1 and therefore need to be transformed into the corresponding quantities in a global frame where the motion of the mixture is tracked. This transformation can be implemented with the help of the components of the three unit vectors, ${\textbf {n}}_x$, ${\textbf {n}}_\rho$, and ${\textbf {n}}_z$, in the global frame, which can be computed from the global coordinates of the center of the sphere and the two ends of the rod. The details of implementing the integrated sphere–rod potential in a molecular dynamics package will be reported in the future.

## Verification of analytical results

The analytical results on the integrated sphere–rod potential, force, and torque, which involve lengthy but compact expressions, are compared to the results from numerically integrating the LJ 12-6 potential for a sphere–rod system. The details of the method adopted for numerical integration are included in the Supplementary Information. In all cases, a perfect agreement is found. Some examples are shown in Fig. 2. The comparison further validates our strategy [Eqs. (9), (10), and (11)] to deal with the special situation where $\rho \sin \theta < a$, i.e., when the central axis of the rod intersects the sphere. In this situation, the integrated potential is still real and finite as long as the rod is outside the sphere. The results presented in Fig. 2 confirm that this is indeed the case if the operations in Eqs. (7) and (8) are adopted.

## Adhesion between a sphere and a thin rod

**Fig. 3:**
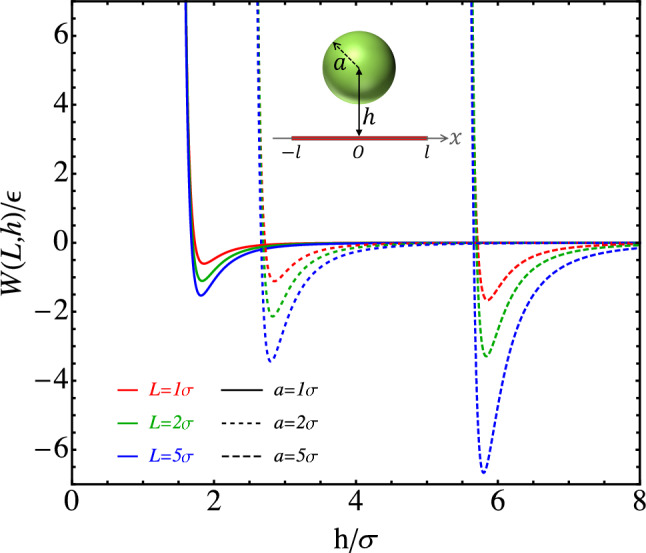
Integrated sphere–rod potential *W*(*h*) vs. *h*, where *h* is the distance between the center of the sphere and the axis of the rod

The integrated sphere–rod potential allows us to investigate the adhesion between a sphere and a slender rod quantitatively. Here we focus on a special arrangement where the center of the sphere is located on the perpendicular bisector of the rod, as shown in the inset of Fig. 3. This configuration is expected to maximize the attraction between the sphere and the rod. The sphere–rod potential is then a function of *h*, which is the distance between the center of the sphere and the axis of the rod, and will be denoted as *W*(*L*, *h*). When $L\rightarrow \infty$, *W*(*L*, *h*) converges to *W*(*h*) in Eq. (15). In this section, we set $\lambda = 1.0\sigma ^{-1}$ and $\rho _s = 1.0\sigma ^{-3}$. In Fig. 3, *W*(*L*, *h*) is plotted against *h* for various values of rod length ($L \equiv 2 l$) and sphere radius (*a*). All curves of *W*(*L*, *h*) vs. *h* are qualitatively similar to that of the LJ 12-6 potential, being attractive at large *h* and becoming repulsive steeply when *h* is reduced below the minimum location of the potential, $h_0$. The data show that $h_0$ is only slightly larger than the radius of the sphere, *a*, and at a given *a*, it is insensitive to *L*. The depth of the potential, $\Delta W \equiv |W(L,h_0)|$, increases as either *a* or *L* increases, as expected. The scaling of the attractive and the repulsive components of the integrated sphere–rod potential with *h* is included in the Supplementary Information.

**Fig. 4:**
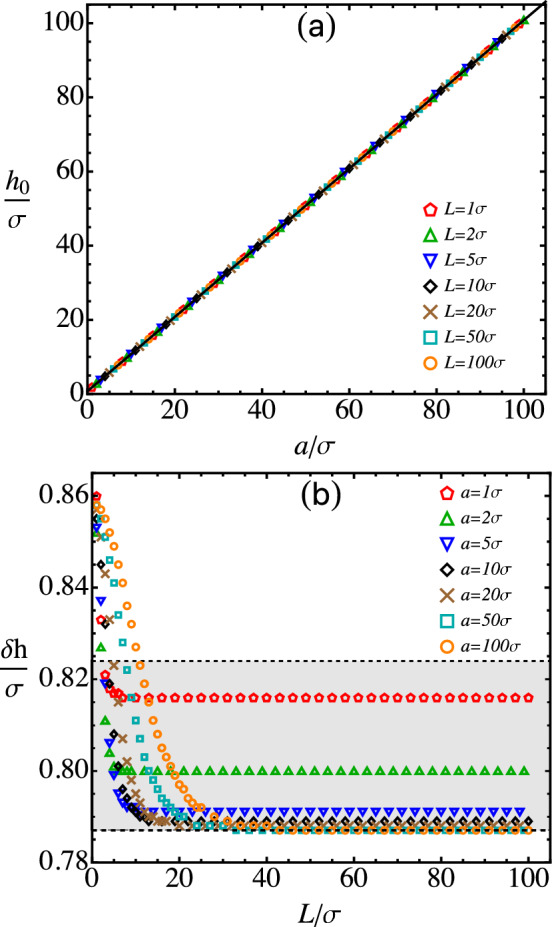
**a** Minimum location ($h_0$) of *W*(*h*) vs. the sphere radius, *a*. **b** Width of the sphere–rod gap ($\delta h \equiv h_0 - a$) vs. the rod length, *L*

The dependence of $h_0$ on *a* and *L* is examined in more detail in Fig. 4. The fact that all data on $h_0$ vs. *a* at various values of *L* are collapsed into a straight line, clearly shown in Fig. 4a, demonstrates that $h_0$ grows linearly with *a* but is almost independent of *L*. In Fig. 4b, the difference between $h_0$ and *a*, defined as $\delta h \equiv h_0 - a$, is plotted against *L* at various values of *a*. As $L\rightarrow 0$, $\delta h$ converges to about $0.86\sigma$. It is noted that when $L\rightarrow 0$ under the constraint of $\lambda L = 1$, the integrated sphere–rod potential is reduced back to the sphere–point potential in Eq. (2). The minimum location of the sphere–point potential can be identified by setting $\frac{d U_\text {SP}(r)}{d r}|_{r=h_0} =0$, which yields the following equation for $h_0$.

25 $$\begin{aligned} \left( h_0 -a \right) ^6 = \frac{2\left( 5 a^6 +27 a^4 h_0^2 +27 a^2 h_0^4 +5 h_0^6\right) \sigma ^6}{5 \left( h_0 + a \right) ^6}.\nonumber \\ \end{aligned}$$

Noting that $h_0$ is only slightly larger than *a*, the right-hand side of Eq. (25) can be approximately evaluated by replacing $h_0$ with *a*, which yields $h_0 = a+ (2/5)^{1/6}\sigma$. Therefore, for the sphere–point potential, $\delta h \simeq 0.858\sigma$. This explains the asymptotic behavior of $\delta h$ in the limit of $L\rightarrow 0$ shown in Fig. 4b.

Figure 4b further shows that as *L* increases, $\delta h$ rapidly decreases to a plateau value that decreases little with increasing *a*. These trends can be understood if we examine the minimum location of the integrated sphere–rod potential in Eq. (15) for an infinite rod. Setting $\frac{d W(h)}{d h}|_{h=h_0} =0$, we obtain the following equation for $h_0$.

26 $$\begin{aligned} \left( h_0 -a \right) ^6 = \frac{11\left( 64 a^6 +432 a^4 h_0^2 +504 a^2 h_0^4 +105 h_0^6\right) \sigma ^6}{800 \left( h_0 + a \right) ^6}~. \end{aligned}$$

Replacing $h_0$ on the right-hand side of Eq. (26) with *a*, we obtain the lower bound of $\delta h$, which is $(3431/10240)^{1/6}\sigma \simeq 0.787\sigma$. On the other hand, for small *a*, $\delta h$ is on the same order as *a*. Therefore, a reasonable upper bound of $\delta h$ can be obtained by replacing $h_0$ on the right-hand side of Eq. (26) with 2*a*, which produces $(5698/18225)^{1/6}\sigma \simeq 0.824\sigma$. These limits are shown as the dashed lines surrounding the gray zone in Fig. 4b, which are consistent with the data on $\delta h$ calculated directly from *W*(*L*, *h*).

For sufficiently large *a* and *L*, $\delta h$ is well approximated by its lower bound, $0.787\sigma$. Therefore, the minimum location of the integrated sphere–rod potential is roughly $h_0 \simeq a + 0.787\sigma$, which corresponds to the straight line in Fig. 4a. Clearly, the data agree with this expression very well.

**Fig. 5:**
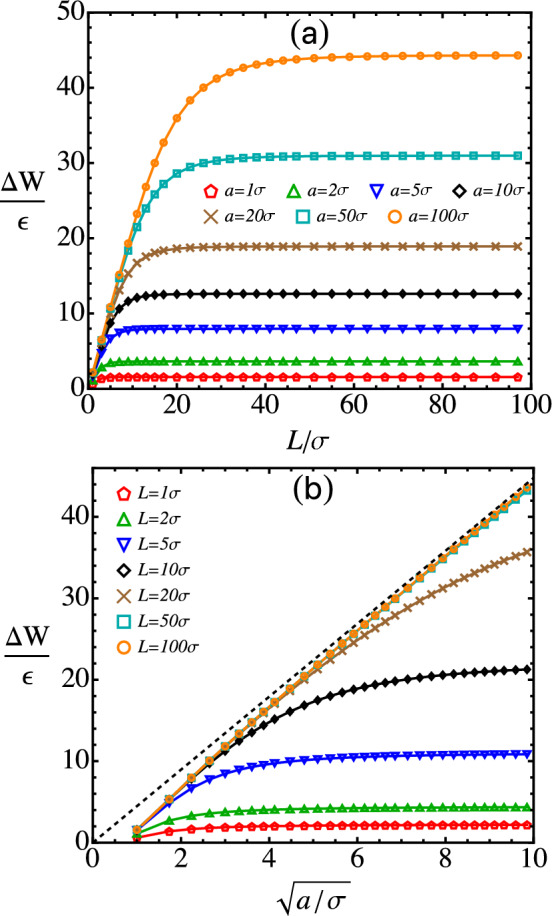
**a** Adhesion between a sphere and a thin rod vs. the rod length (*L*) at various values of the sphere radius (*a*). **b** Adhesion vs. the square root of the sphere radius at various rod lengths; the dashed line indicates $\Delta W = 4.48 \epsilon \sqrt{a/\sigma }$

The adhesion ($\Delta W$) between a sphere and a rod can be defined as the depth, $|W(L,h_0)|$, of the integrated sphere–rod potential. $\Delta W$ is plotted against the length of the rod (*L*) for spheres of various sizes in Fig. 5a. In all cases, $\Delta W$ first increases with increasing *L*, but then quickly reaches a plateau as *L* exceeds a threshold $L_t$. Although $L_t$ increases with increasing *a*, the ratio, $L_t/a$, gradually decreases as the sphere becomes larger.

The adhesion between a sphere and an infinite rod can be obtained from $W(h_0)$ with Eq. (15). Based on the preceding discussion of Fig. 4, $h_0 - a\simeq 0.787\sigma$ for a sufficiently large sphere ($a \gtrsim 10\sigma$). Then Eq. (15) implies that the adhesion is proportional to the square root of the sphere size, i.e.,

27 $$\begin{aligned} \Delta W \equiv |W(h_0)|\simeq 4.48 \epsilon \sqrt{a/\sigma }~, \end{aligned}$$

for $\lambda = 1.0\sigma ^{-1}$ and $\rho _s = 1.0\sigma ^{-3}$. This scaling agrees with the data presented in Fig. 5b, where $\Delta W$ is plotted against $\sqrt{a}$. For short rods, $\Delta W$ increases with *a* but eventually plateaus. The plateau value increases as the length of the rod increases. For long rods (e.g., $L \gtrsim 50\sigma$) and sufficiently large spheres, the curve of $\Delta W$ vs. $\sqrt{a}$ conforms to the scaling relation in Eq. (27), which is indicated by the dashed line in Fig. 5b.

## Discussion

Although we focus on the integrated form of the LJ 12-6 potential for a sphere–thin rod system here, the method can be used to integrate a more general LJ 2*n*-*n* potential, which reads [36]

28 $$\begin{aligned} U_\text {2n-n}(r) = 4\epsilon \left[ \left( \frac{\sigma }{r}\right) ^{2n} - \left( \frac{\sigma }{r}\right) ^n \right] ~, \end{aligned}$$

with *n* being a whole number. Only the case with $n=6$ is discussed in detail here, but Ostrogradsky’s method can be used to integrate any rational functions [23, 24]. Using the coordinate system defined in Fig. 1, we have $r=\sqrt{y^2+h^2}$, and both $1/r^{2n}$ and $1/r^n$ are integrable rational functions as long as *n* is even. Vliegenthart et al. have studied the phase behavior, structure, and dynamical properties of LJ systems with $n=6$, 11, 12, and 18, among which three are even integers [36]. The corresponding LJ 2*n*-*n* potentials in principle can be integrated for a sphere and a thin rod and expressed in terms of common functions, through the method used here. As *n* increases, the range of the LJ 2*n*-*n* potential decreases, making it transition from a long-range potential to a very short-range one. Therefore, the integrated form of the LJ 2*n*-*n* potential may be useful for understanding the behavior of various rod-sphere mixtures that possess interactions with different spatial ranges.

## Conclusions

We present accurate analytic expressions for the integrated Lennard-Jones (LJ) 12-6 potential between a sphere and a thin rod in arbitrary three-dimensional configurations, with the two objects modeled as continuous media of LJ material points. The results are expressed in compact analytical forms. The expressions for force and torque are also presented.

The integrated sphere–rod potential can be used for theoretical descriptions and computational modeling of soft matter systems involving mixtures of cylindrical and spherical objects [25–30]. For example, the potential is applied to investigate the adhesion between a sphere and a thin rod. In the equilibrium configuration where the integrated potential is minimized, the center of the sphere is located on the perpendicular bisector of the rod. For a sufficiently long rod and a sufficiently large sphere, the equilibrium gap between the two is found to have an almost constant width, approximately $0.787\sigma$, where $\sigma$ is the length unit defined in the LJ potential. In this situation, the adhesion between the sphere and the rod is found to scale with $\sqrt{a}$, where *a* is the radius of the sphere. This scaling is expected to hold as long as the attraction between the sphere and the rod is dominated by van der Waals interactions. This interesting scaling prediction has yet to be confirmed with experiment.

## Supplementary Information

Below is the link to the electronic supplementary material.

Supplementary file 1 (pdf 711 KB)

## Data Availability

The data that support the findings of this study are available from the corresponding author upon reasonable request.
