# Supplementary material for: Analytical sphere–thin rod interaction potential
Source: Eur Phys J E Soft Matter. 2025 Apr 7;48(4-5):15. doi: 10.1140/epje/s10189-025-00480-9 (PMC11976830; doi:10.1140/epje/s10189-025-00480-9)
Supplement: Supplementary file 1 — (pdf 711 KB) [file 10189_2025_480_MOESM1_ESM.pdf]

Supplementary Information for "Analytical Sphere-Thin Rod Interaction Potential"

Junwen Wang[1,2,3] and Shengfeng Cheng[4,2,3,1,*]

[1] *Department of Mechanical Engineering, Virginia Tech, Blacksburg, Virginia 24061, USA*
[2] *Center for Soft Matter and Biological Physics, Virginia Tech, Blacksburg, Virginia 24061, USA*
[3] *Macromolecules Innovation Institute, Virginia Tech, Blacksburg, Virginia 24061, USA*
[4] *Department of Physics, Virginia Tech, Blacksburg, Virginia 24061, USA*

* chengsf@vt.edu

S1. CONTINUITY OF THE INTEGRATED SPHERE-ROD POTENTIAL

In the main text, formulae are provided for the integrated sphere-rod potential at $h > a$, $h = a$ and $h < a$, respectively, where $h = \rho \sin\theta$ is the distance from the center of the sphere to the axis of the rod, and a is the radius of the sphere (see Fig. 1 of the main text for the definition of the coordinate system and symbols). The continuity of these formulae is demonstrated in Figs. S1 and S2 here, where either ρ or θ is varied, while the other is fixed, to change h from a value smaller than a to a value larger than a. The results clearly show that the analytical formulae presented in the main text are continuous at $h = a$.

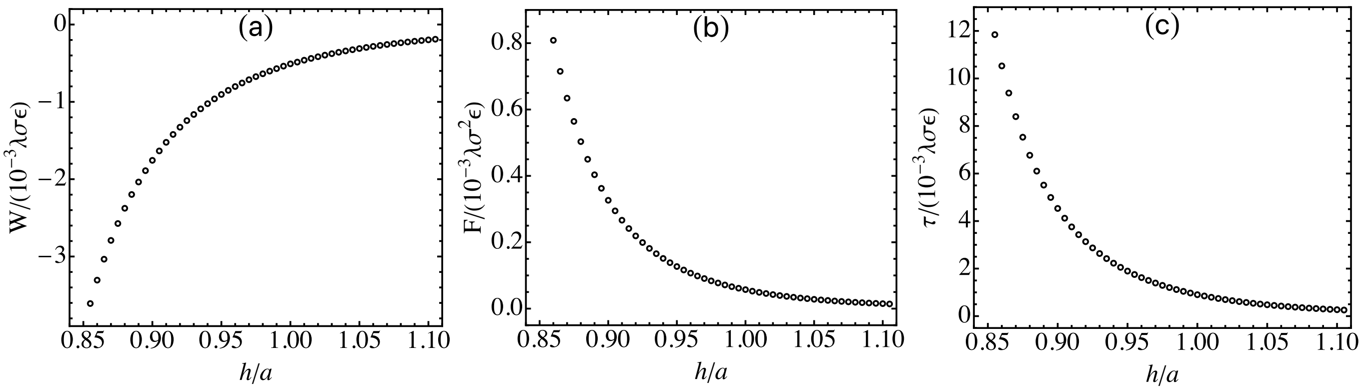

FIG. S1. Integrated (a) potential, (b) force, and (c) torque between a sphere and a rod at $a = 10\sigma$, $L = 5\sigma$, $\theta = \pi/6$, and $h = \rho \sin\theta$ with ρ varying from 17σ to 22σ.

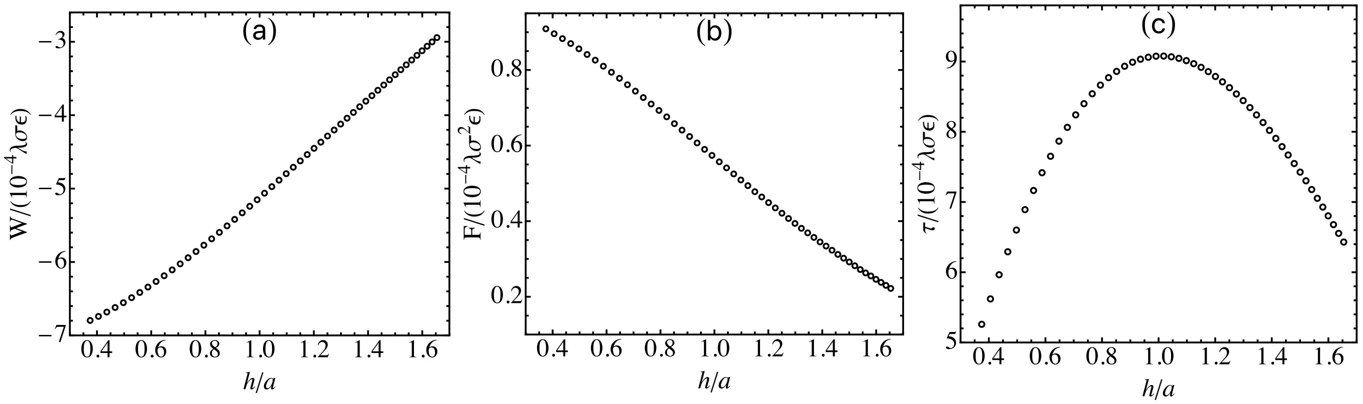

FIG. S2. Integrated (a) potential, (b) force, and (c) torque between a sphere and a rod at $a = 10\sigma$, $L = 5\sigma$, $\rho = 20\sigma$, and $h = \rho \sin\theta$ with θ varying from $\pi/20$ to $3\pi/10$.

S2. SCALING OF THE ATTRACTIVE AND REPULSIVE COMPONENTS OF THE INTEGRATED SPHERE-ROD POTENTIAL

The Lennard-Jones 12-6 potential [Eq. (1) in the main text] has an attractive component (the $1/r^6$ term) and a repulsive component (the $1/r^{12}$ term). Similarly, the integrated sphere-point potential and the integrated sphere-rod potential each have two components, one attractive (W_a, which is negative) and one repulsive (W_r, which is positive). To understand how each component varies with separation, it is convenient to rewrite the integrated sphere-point potential [Eq. (2) in the main text] as

$$U_{\mathrm{SP}}(r) \;=\; \frac{2\rho_s a^3 \sigma^6 A_{cs}}{9}\left[\frac{\sigma^6}{(r^2-a^2)^6} + \frac{36a^2\sigma^6}{5\,(r^2-a^2)^7} + \frac{72a^4\sigma^6}{5\,(r^2-a^2)^8} + \frac{128a^6\sigma^6}{15\,(r^2-a^2)^9} - \frac{1}{(r^2-a^2)^3}\right] . \tag{S1}$$

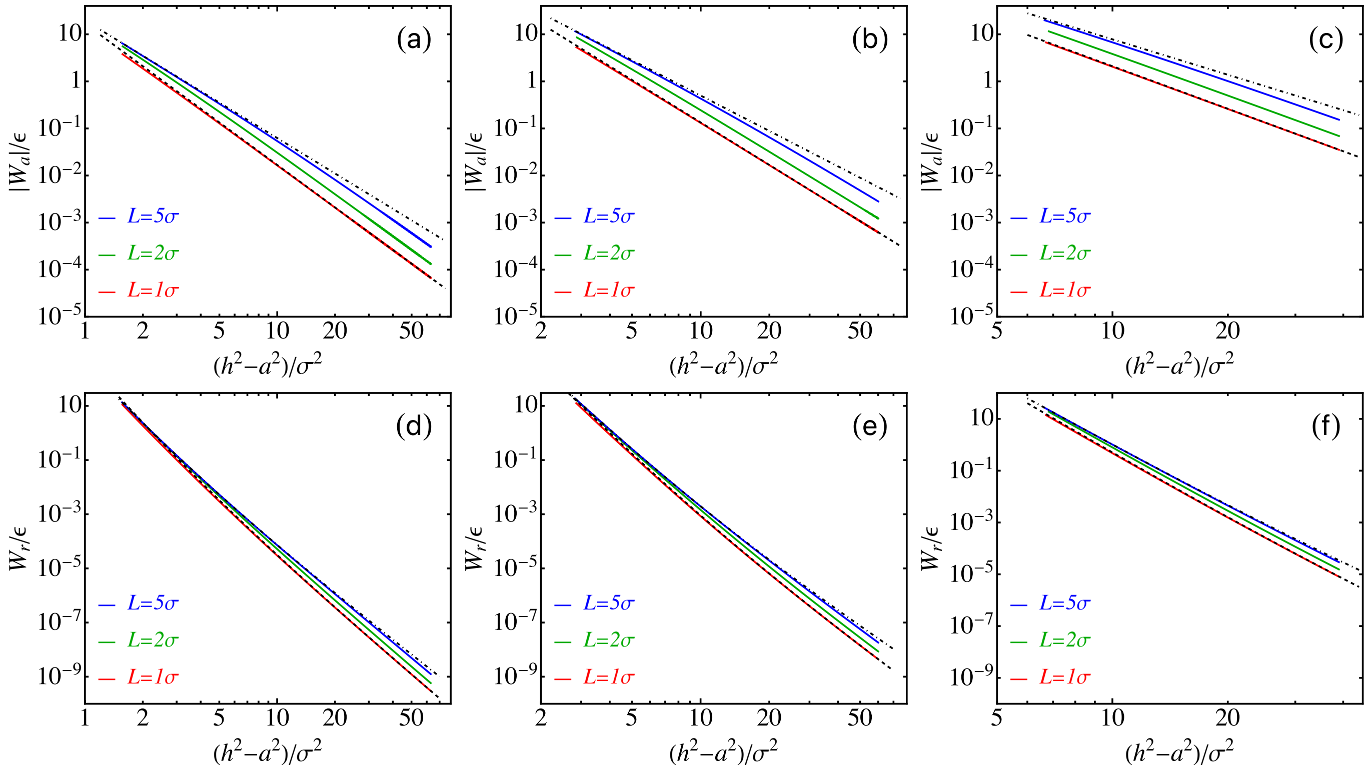

FIG. S3. Integrated attraction [$|W_a|$, in (a)-(c)] and repulsion [W_r, in (d)-(f)] vs. $h^2 - a^2$ on a log-log scale for $a = 1\sigma$ [(a) and (d)], $a = 2\sigma$ [(b) and (e)], and $a = 5\sigma$ [(c) and (f)] at various lengths of the rod. The dashed line represents the attractive component [in (a)-(c)] or the repulsive component [in (d)-(f)] of the integrated sphere-point potential. The dashdotted line represents the attractive component [in (a)-(c)] or the repulsive component [in (d)-(f)] of the integrated potential between a sphere and an infinite rod.

The first four terms in the summation in Eq. (S1) represent the repulsion while the last term is the attraction. Both components vary as (a sum of) negative powers of $r^2 - a^2$. Furthermore, the integrated potential between a sphere and an infinite rod [Eq. (15) in the main text] can be rewritten as

$$W(h) = \frac{\pi\lambda\rho_s a^3\sigma^6 A_{cs}}{3}\left[\frac{21\sigma^6}{128\,(h^2-a^2)^{11/2}} + \frac{693a^2\sigma^6}{640\,(h^2-a^2)^{13/2}} + \frac{1287a^4\sigma^6}{640\,(h^2-a^2)^{15/2}} + \frac{143a^6\sigma^6}{128\,(h^2-a^2)^{17/2}} - \frac{1}{4\,(h^2-a^2)^{5/2}}\right]. \tag{S2}$$

Again, the first four terms in the summation represent the repulsive contribution, while the last term is the attractive component. Both repulsion and attraction vary as (a sum of) negative powers of $h^2 - a^2$.

Here we focus on the special configuration studied in Sec. IV of the main text, where the center of the sphere is located on the perpendicular bisector of the rod. In this case, the integrated sphere-rod potential is a function of h, the distance of the center of the sphere from the axis of the rod, with the radius (a) of the sphere and the length (L) of the rod as parameters. In Fig. S3, the attractive (W_a) and the repulsive (W_r) parts of the integrated sphere-rod potential are separated and plotted against $h^2 - a^2$ on a log-log scale. Figs. S3(a)-(c) show that when the rod's length $L \to 0$, the attractive component approaches the attractive term of the integrated sphere-point potential, while the curve of attraction at a large value of L is close to the attractive term of the integrated potential between the sphere and an infinite rod. The similar trends are also reflected in repulsion, as shown in Figs. S3(d)-(f).

S3. NUMERICAL INTEGRATION METHOD

In this section, we provide details on the calculation of potential, force, and torque via numerical integration. The integrated potential involves the numerical integration of the sphere-point potential along the length of the rod, which

is carried out using Python's `quad` function from the `scipy.integrate` module. It employs an adaptive quadrature method to achieve high accuracy.

The adaptive quadrature technique used by `quad` dynamically adjusts the number and placement of integration points according to the behavior of the function being integrated. This method ensures that regions where the function exhibits rapid changes, steep gradients, or near-singularities, are sampled more densely, while relatively flat regions of the function are sampled less frequently.

Algorithm 1 Adaptive Integration Procedure

```
1: procedure INTEGRATE(f, a, b, τ)
2:     Q ≈ ∫_a^b f(x) dx
3:     ε ← |Q − ∫_a^b f(x) dx|
4:     if ε > τ then
5:         m = (a+b)/2
6:         Q = INTEGRATE(f, a, m, τ/2) + INTEGRATE(f, m, b, τ/2)
7:     return Q
```

In the adaptive integration algorithm, an approximation (Q) to the integral of $f(x)$ over the interval $[a, b]$ is computed (line 2), as well as an estimate of error (ε in line 3). If the estimated error is larger than the required tolerance (τ in line 4), the interval is subdivided (line 5), and the quadrature procedure is applied separately to each divided region (line 6). In the end, the procedure returns (line 7) either the initial estimate or the sum of the recursively computed values in the divided regions.

For the force, the calculation starts by expressing the differential force acting on an infinitesimal segment of the rod. This differential force is defined as a function of the segment's position along the rod. The integration is then performed for each component of the force along the entire length of the rod using `quad`. The adaptive quadrature technique ensures that regions where the force function exhibits rapid variations (e.g. near the closest separation between the sphere and the rod) are evaluated with finer resolution, while smoother regions are computed with fewer function evaluations.

For the torque, a similar approach is used. The torque on each segment of the rod is calculated as the cross product of the position vector of the segment, with respect to the center of the rod, and the differential force acting on that segment. The differential torque, expressed as a function of the position along the rod, is then integrated over the entire length of the rod. The `quad` function in Python adapts to the complexity of the torque function, ensuring accurate computation even when the arrangement of the sphere and the rod and the resulting force distribution lead to nonlinearity.
